# Suppression of polyi:c-inducible gene expression by EP3 in murine conjunctival epithelium

**DOI:** 10.1186/2045-7022-4-S2-P58

**Published:** 2014-03-17

**Authors:** Mayumi Ueta, Katsura Mizushima, Yuji Naito, Shuh Narumiya, Shigeru Kinoshita

**Affiliations:** 1 Kyoto Prefectural University of Medicine, Doshisha University, Doshisha University, Department of Ophthalmology, Kyoto, Japan; 2 Kyoto Prefectural University of Medicine, Molecular Gastroenterology and Hepatology, Kyoto, Japan; 3 Kyoto University, Department of Pharmacology and Faculty of Medicine, Kyoto, Japan; 4 Kyoto Prefectural University of Medicine, Department of Ophthalmology, kyoto, Japan

## 

We previously reported that EP3, a subtype of prostaglandin E2 receptors, negatively regulates eosinophilic infiltration in murine experimental allergic conjunctivitis induced by TLR3, which causes reduced eosinophilic conjunctival inflammation in TLR3/EP3 double knock-out (DKO) mice although in EP3-KO mice eosinophilic conjunctival inflammation is pronounced. We also documented that in human conjunctival epithelial cells, the EP3 agonist suppressed the production of cytokines such as CXCL10, CXCL11, IL6, CCL5, TSLP, and MCP-1 induced by polyI:C, a TLR3 ligand. EP3 was dominantly expressed in conjunctival epithelial cells, airway epithelial cells, and keratinocytes. To examine the effects of EP3 against polyI:C-inducible gene expression in conjunctival epithelium we performed gene expression analysis of the polyI:C-stimulated conjunctival epithelium in wild-type, EP3-KO-, and EP3/TLR3 DKO mice. Using GeneChip® we first examined the comprehensive effects of gene expression in polyI:C-stimulated conjunctival epithelium of wild-type mice. We found that after 6-hr stimulation, 31 transcripts were up-regulated more than 10-fold. Quantitative RT-PCR confirmed that 21 of the 31 transcripts were significantly (> 3-fold) up-regulated. Next, to identify the transcripts regulated by EP3 we compared the gene expression of these 21 transcripts in polyI:C stimulated conjunctival epithelium of wild-type and EP3-KO mice by quantitative RT-PCR. We found that all 21 transcripts were expressed significantly stronger in polyI:C stimulated conjunctival epithelium of EP3-KO mice. We also confirmed that the mRNA expression of these 21 transcripts was significantly reduced in polyI:C stimulated conjunctival epithelium of EP3/TLR3 DKO- compared to EP3- KO mice. GeneChip® analysis also showed that the number of 4 transcripts was more than 5 times greater in polyI:C stimulated conjunctival epithelium of EP3-KO- than wild-type mice although in wild-type mice these 4 transcripts were not significantly up-regulated after 6-hr polyI:C stimulation. Quantitative RT-PCR confirmed that the number of 2 of the 4 transcripts was more than 100-fold higher in polyI:C stimulated EP3 KO- than wild-type mice. In summary, we found that EP3 suppressed polyI:C, a TLR3 ligand, inducible genes in polyI:C stimulated murine conjunctival epithelium. Our findings suggest that EP3 and TLR3 in conjunctival epithelium play a critical role in regulating ocular surface inflammation.

